# Precipitation Behavior of Mg-7Gd-3Y-2Zn-0.5Zr Alloy during Isothermal Aging

**DOI:** 10.3390/ma14071737

**Published:** 2021-04-01

**Authors:** Jiawei Yuan, Ting Li, Kui Zhang, Xinggang Li, Yongjun Li, Minglong Ma, Guoliang Shi, Zhiwei Du, Wei Liu, Yonggang Peng

**Affiliations:** 1 State Key Laboratory of Non-ferrous Metals and Processes, GRIMAT Engineering Institute Co., Ltd., No.11, Xinkedong Street, Huairou District, Beijing 101407, China; yuanjiawei@grinm.com (J.Y.); zhkui@grinm.com (K.Z.); lxg1218@grinm.com (X.L.); lyj@grinm.com (Y.L.); maminglong@grinm.com (M.M.); shiguoliang@grinm.com (G.S.); 2 Guobiao (Beijing) Testing & Certification Co., Ltd., No.2, Xinjiekouwai Street, Xicheng District, Beijing 100088, China; duzhiwei@gbtcgroup.com (Z.D.); liuwei@gbtcgroup.com (W.L.); pengyonggang@gbtcgroup.com (Y.P.)

**Keywords:** magnesium alloy, precipitation, LPSO

## Abstract

Precipitate phases in an Mg–7Gd–3Y–2Zn–0.5Zr alloy aged isothermally at 240 °C were examined using high-resolution transmission electron microscopy (TEM) and high-angle annular dark-field scanning TEM. The two types of precipitation sequence that involve Mg–Gd and long period stacking ordered (LPSO) type were found. The LPSO type sequence consisted of the precipitation of γ′′, γ′, 14H-LPSO/18R-LPSO. The Mg–Gd type precipitation sequence involved the formation of β′(b.c.o.) and β1(f.c.c.). The sequence, morphology, distribution, and crystal structure of these precipitates formed during isothermal aging were investigated. The results indicated that the priority precipitation of Mg–Gd and LPSO type sequences during aging can be affected by Nd, which has a higher diffusion coefficient than Gd and Y. The dislocation structures and strengthening mechanism were also discussed.

## 1. Introduction

Magnesium alloys are promising materials for automobiles, aircrafts, 3C products, and many other industrial fields, due to their low destiny and high specific strength [1,2,3,4,5,6]. Among these alloys, rare earth-containing magnesium alloys, such as Mg–Gd and Mg–Gd–Y, have received considerable attention because they harden at precipitation. This property results in high specific strength at both room, and elevated, temperatures [7,8,9,10]. In Mg–Gd-based alloys, the precipitation sequences can be proposed as follows: α-Mg(S.S.S.S)→β″→β′→β_1_→β(f.c.c,Mg_5_Gd), in which β′ and β_1_ are the key strengthening precipitate phases [11,12,13,14]. The crystallography characterizations of these precipitates have been widely reported.

Recently, the addition of Zn in Mg–Gd series alloys has been demonstrated to promote the formation of a long period stacking ordered (LPSO) structure. This structure can improve both strength and ductility simultaneously [15,16,17,18]. The precipitation of the LPSO-type sequence during isothermal aging involves the formation of the metastable phases γ″ and γ′, as reported by Nie and Wei et al. [19,20,21,22]. The γ″ phase forms because (0001)α plates with a thickness of a single unit cell height and has an ordered hexagonal structure (space group P$\bar{6}$2m, *a* = 0.560 nm, *c* = 0.444 nm). The γ′ phase also forms on (0001)α, but has a large aspect ratio. The crystal structure of γ′ is disordered hexagonal, with a P$\bar{3}$m1 space group (*a* = 0.321 nm, *c* = 0.781 nm).

In Mg–RE–Zn alloys, the composite precipitation of the β′ and γ′ phases enhances the mechanical properties of strength and ductility, as reported by many other studies [23,24]. Wei et al. [19] found that the composite β′ and γ′ precipitates, which have a relative perpendicular distribution, effectively strengthened the grain interior and hindered twining during tensile tests. Xu investigated the precipitation behavior of β′, β_1_, stacking faults, and 14H LPSO phase during aging heat treatment in a Mg–Gd–Y–Zn–Zr alloy [25]. These results show that two types of precipitation sequence occur simultaneously during aging, and that the precipitation behavior is complicated in Mg–RE–Zn alloys. However, the precipitation sequence, phase transformation, and interaction between precipitates in these two types of precipitation sequence have not yet been elaborated systematically.

In this paper, the crystallographic characterization, especially the precipitation sequence, phase transformation, and interactions, between the two types of precipitates in a Mg-7Gd-3Y-2Zn-0.5Zr alloy aged isothermally at 240 °C was analyzed using high-resolution transmission electron microscopy (HRTEM) and high-angle annular dark-field scanning transmission electron microscopy (HAADF-STEM).

## 2. Materials and Methods

An alloy with the nominal composition of Mg-7Gd-3Y-2Zn-0.5Zr was prepared by melting pure Mg (99.99%), Gd (99.99%), Y (99.99%), Zn (99.99%), and Mg-30 wt% Zr master alloy in a high-frequency induction melting furnace under the protection of Ar and C_2_H_2_F_2_ atmosphere. The alloy was cut into small pieces and solution-treated at 520 °C for 24 h in a muffle furnace. After being solution-treated, the samples were quenched immediately by room temperature water and then were aged at 240 °C. The hardness measurement was carried out using a Brinell hardness tester; the test load head and dwell time were 30 kgf and 25 s, respectively. Each specimen was tested at five evenly spaced locations. Microstructure observations were performed through scanning electron microscopy (SEM, JEOL, Tokyo, JPN) equipped with Energy Dispersive Spectroscopy (EDS,) and transmission electron microscopy (TEM, Thermofisher, Waltham, MA, USA). Then, 3-mm diameter disc samples for TEM, HRTEM, and HAADF-STEM analyses were ground to a thickness of 60 μm and subjected to ion beam thinning by PIPS 695, GATAN, Pleasanton, CA, USA). The microstructures were observed and analyzed by JEM-2010 JEOL, Tokyo, JPN and FEI F20 electron microscopes (Tecnai, Hillsboro, OR, USA) operated at 200 KV.

## 3. Results

### 3.1. As-Cast and As-Homogenized Microstructures

**Figure 1:**
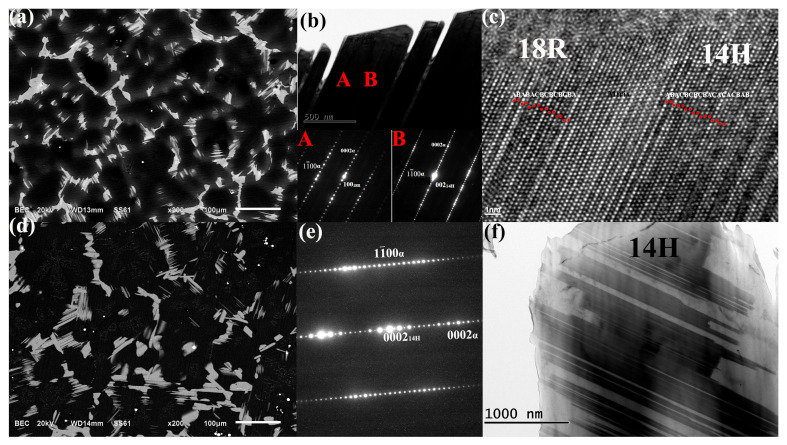
Microstructure for as-cast alloy: (**a**) SEM image; (**b**) bright-field image of the long period stacking ordered (LPSO) phase and corresponding SAED pattern for zones A and B taken along ${\left[11\bar{2}0\right]}_{\alpha }$; (**c**) high-resolution transmission electron microscopy (HRTEM) image for LPSO in Figure 1b taken along the ${\left[11\bar{2}0\right]}_{\alpha }$ zone axis; the microstructure for as-homogenized alloy: (**d**) SEM image; (**e**) SAED pattern for the LPSO phase taken along the ${\left[11\bar{2}0\right]}_{\alpha }$ zone axis; and (**f**) corresponding bright-field image for the LPSO phase.

Figure 1 shows the as-cast and as-homogenized microstructure of Mg-7Gd-3Y-2Zn-0.5Zr alloy. Figure 1a shows the block-shaped phases distributed along grain boundaries. To further understand the crystal characteristics of these phases, we conducted TEM and HRTEM. Figure 1b shows the two adjacent areas marked by A and B with different structures. The selected area electron diffraction (SAED) pattern acquired from the ${\left[\bar{1}\bar{1}20\right]}_{\alpha }$ zone axis shows 13 extra spots between the transmitted beam and the (0002)*_α_* fundamental reflection. This finding indicates the 14H-type LPSO with a lattice parameter of *a* = 1.112 and *c* = 3.647 nm. The orientation relationship between the 14H-type LPSO structure and matrix can be described as ${\left(0001\right)}_{14H}$//${\left(0001\right)}_{\alpha }$, ${\left[01\bar{1}0\right]}_{14H}$//${\left[\bar{1}\bar{1}20\right]}_{\alpha }$. The SAED pattern for B indicates that the distance between the transmitted beam and (0002)*_α_* fundamental reflection was divided by 6, which indicated the 18R-type LPSO with a lattice parameter of *a* = 1.112, *b* = 1.926, and *c* = 4.689, and β = 83.25°. Figure 1c shows the HRTEM image of LPSO phase and the stacking sequences of ABABACBCBCBCBA (14H-type) and ABACBCBCBACACACBAB (18R-type), with periods of 3.64 and 4.68 nm for A and B, respectively. The HRTEM image also shows that A and B were separated and joined by a Mg matrix with a stacking sequence of ABABAB.

After homogenization heat treatment, Figure 1d shows that the volume fraction of LPSO phase decreased compared with that in Figure 1a, but these phases did not completely dissolve into the matrix. The SAED pattern shown in Figure 1e indicates that the structure of the residual LPSO was 14H type. Thus, 18R LPSO phase replacement by 14H LPSO could be deduced during the homogenization heat treatment. 

### 3.2. Age Hardening Behavior

**Figure 2:**
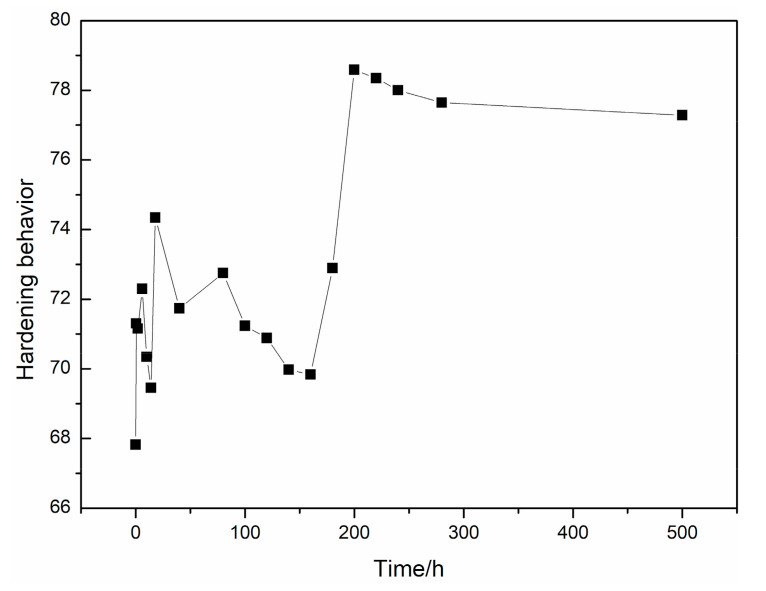
Age hardening curve for alloy aged at 240 °C.

The age hardness curve for the as-homogenized alloy isothermal aging at 240 °C is shown in Figure 2. During the initial stage, from 0 h to 180 h, an increase in hardness is not evident, and only a slight increase can be observed, the maximum hardness value is only 74 HB. The aging time to reach peak hardness was about 200 h, which is much longer than that for many other Mg–RE–Zn alloys. The peak aging hardness was 79 HB, and with the aging time extended to 500 h, the hardness of the alloy decreased slightly. The different hardening behaviors suggest that the main strengthening phase may be differential. To further understand the precipitates in this alloy, microstructures after aging at 240 °C for 18, 200, and 500 h were analyzed.

### 3.3. As-Aged Microstructures

#### 3.3.1. Under-Aged Microstructures: $\gamma^{\prime \prime }$ Precipitates

**Figure 3:**
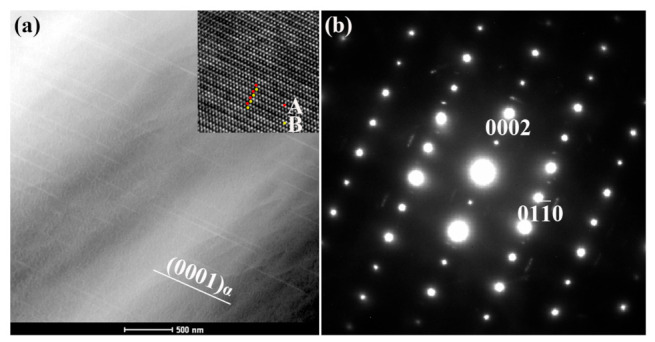
(**a**) High-angle annular dark-field (HAADF) image of the sample aged for 18 h, and the corresponding HRTEM image; and (**b**) SAED pattern taken along ${\left[\bar{2}110\right]}_{\alpha }$ for the $\gamma^{\prime \prime }$ phase.

Figure 3a shows the HAADF-STEM image of alloy aging at 240 °C for 18 h to reveal the microstructure of the initial aging stage, and in which a slight increase in hardness can be observed. When viewed along the ${\left[2\bar{1}\bar{1}0\right]}_{\alpha }$ zone axes, a few needle-like precipitates that are parallel to the basal ${\left(0001\right)}_{\alpha }$ plane can be found. The length of these needle-like precipitates is approximately several hundred nanometers. Given that the intensity of the HAADF-STEM image is approximately proportional to the square of Z, the HAADF-STEM observation reveals that solute elements, such as Zn and Gd, Y concentrate in these needle-like structures. However, the SAED pattern acquired along the ${\left[2\bar{1}\bar{1}0\right]}_{\alpha }$ zone axes shows that no other extra spots, except the matrix, can be found. This finding indicates that no stacking fault was included in the precipitates. Further HRTEM analysis for the precipitates is shown in the inserted image, and a similar stacking sequence of ABABAB can be found. Nie reported that this microstructural feature may be consistent with $\gamma^{\prime \prime }$, and the $\gamma^{\prime \prime }$ precipitate is composed of three ${\left(0001\right)}_{\alpha }$ atomic layers enriched by heavy atoms, and no stacking faults are induced. The $\gamma^{\prime \prime }$ phase has an ordered hexagonal structure (space group P$\bar{6}$2m, *a* = 0.560 and *c* = 0.444 nm). The orientation relationship between $\gamma^{\prime \prime }$ and the matrix was ${\left(0001\right)}_{\gamma^{\prime \prime }}$ // ${\left(0001\right)}_{\alpha }$ and ${\left[10\bar{1}0\right]}_{\gamma^{\prime \prime }}$//${\left[2\bar{1}\bar{1}0\right]}_{\alpha }$. Thus, during the initial aging stage, several $\gamma^{\prime \prime }$ precipitated from the matrix.

#### 3.3.2. Peak-Aged Microstructures: $\gamma^{\prime }$+β′/β1 Precipitates

**Figure 4:**
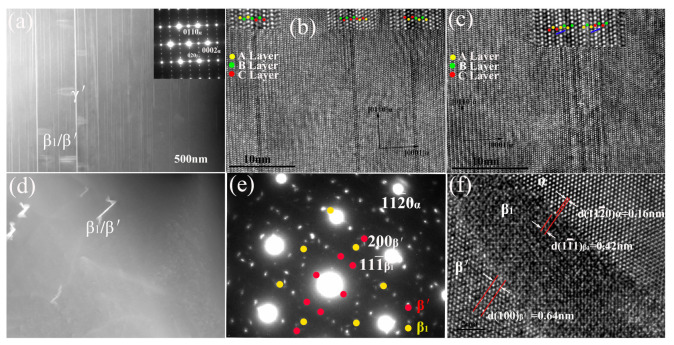
Microstructure for alloy aged for 200 h: (**a**) HAADF image; (**b**), (**c**) HRTEM images taken along ${\left[\bar{2}110\right]}_{\alpha }$; (**d**) bright-field image; (**e**) SAED pattern; and (**f**) HRTEM image taken along ${\left[0001\right]}_{\alpha }.$

As indicated in the hardness curve, the aging time to reach peak hardness was about 200 h, and the peak-aged plateau was from 200 h to 500 h. This long period of peak aging for the plateau suggests the good thermal stability of the main strengthening phases. Figure 4a shows the HAADF-STEM image and corresponding SAED patterns of the alloy aged at 240 °C for 200 h. The two types of precipitates, which are vertical to each other, can be viewed from the ${\left[2\bar{1}\bar{1}0\right]}_{\alpha }$ zone axes. Figure 4a shows that the several precipitates rich in heavy atoms that are parallel to basal ${\left(0001\right)}_{\alpha }$ plane have a length of several micrometers. However, in contrast to the $\gamma^{\prime \prime }$ phase in the alloy at 240 °C for 18 h, the strong streaks that appeared along the ${\left[0001\right]}_{\alpha }$ direction corresponding to these basal ${\left(0001\right)}_{\alpha }$ phases in the SAED pattern indicate that stacking faults are included. The HRTEM for these ${\left(0001\right)}_{\alpha }$ plates indicates that many types of precipitates that consist of diverse numbers of closely packed layers were formed in the matrix. Some precipitates consist of a single building block, and the stacking sequence of ABABCBC and enrichment of heavy elements in two atomic layers satisfy the feature of the $\gamma^{\prime }$ phase [20,22]. Figure 4c shows that some plates consisting of several building blocks can also be found, suggesting that the growth of $\gamma^{\prime }$ phase along ${\left[0001\right]}_{\alpha }$ also occurred with prolonged aging time. Figure 4c shows six layers of ${\left(0001\right)}_{\alpha }$ Mg between the two adjacent $\gamma^{\prime }$ precipitates, but not any state type of LPSO. 

To carefully examine the characterization of precipitates that formed perpendicular to ${\left(0001\right)}_{\alpha }$, we recorded the TEM image along the ${\left[0001\right]}_{\alpha }$ zone axes, as shown in Figure 4d. The two types of particles that intergrew with each other can be found, and the size of these precipitates was approximately 100–200 nm. The overlapped SAED patterns for the precipitates in Figure 4e are indexed in accordance with the b.c.o. structure with *a* = 0.64, *b* = 2.22, and *c* = 0.52 nm, and the f.c.c. structure with *a* = 0.74 nm indicates that these precipitates are β′ and β_1_ phases. The orientation relationship between β′, β_1_, and the matrix can be described as $[1\bar{1}0]\;\beta_{1}//[001]\beta^{\prime }//[0001]\alpha$, $(11\bar{1})\;\beta_{1}//(100)\beta^{\prime }//(11\bar{2}0)\alpha$. The HRTEM image shown in Figure 4f indicates a semi-coherent relationship between β_1_ and the matrix.

#### 3.3.3. Over-Aged Microstructures: γ′ + LPSO(14H/18R) + β′/β1 Precipitates

**Figure 5:**
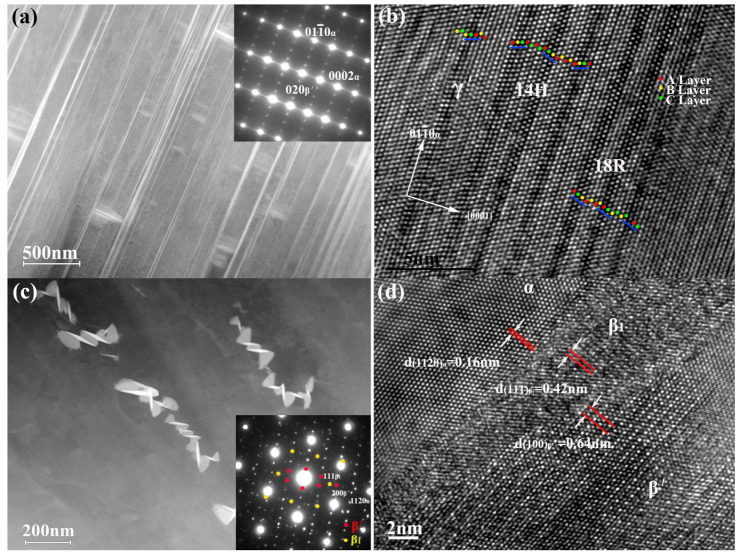
Microstructure for alloy aged for 500 h: (**a**) HAADF image and (**b**) HRTEM image taken along $\left[\bar{2}110\right]$*α* (**c**) HAADF image, and (**d**) HRTEM image taken along ${\left[0001\right]}_{\alpha }.$

The microstructure after 500 h of aging is shown in Figure 5. As viewed from the ${\left[2\bar{1}\bar{1}0\right]}_{\alpha }$ zone axes, strong streaks that appear along the ${\left[0001\right]}_{\alpha }$ direction indicate the existence of the $\gamma^{\prime }$ phase. However, unlike the precipitation morphology of peak-aged alloy, the growth of $\gamma^{\prime }$ phase along the [0001] direction can be found. To examine the detailed structure of these grown $\gamma^{\prime }$ phases, we employed HRTEM, as shown in Figure 5b. Some precipitates consist of a single building block, and the stacking sequence of ABABCBC indicates the existence of the $\gamma^{\prime }$ phase. Some plates consist of several building blocks, and long-period atomic stacking sequence along the [0001]α direction can be measured as ABABCACACACBABA and ACACBABABACBCBCBAC. In one type of phase, the building blocks “ABCA” and “ACBA” are arranged in the opposite shear direction, with three matrix layers between the two blocks. This finding is consistent with the characterization of 14H-LPSO. However, in the other type of phase, the building blocks “ACBA,” “BACB,” and “CBAC” are arranged in the same shear direction, with two matrix layers between the two adjacent building blocks. These three building blocks with the matrix layers, together constitute 18R-LPSO. Thus, the $\gamma^{\prime }$ phase had partially evolved to 14H and 18R LPSO phases during the over-aged period.

The morphology and corresponding SAED pattern for β′/β_1_ precipitates viewed from ${\left[0001\right]}_{\alpha }$ zone axes are shown in Figure 5c. The size of the β′/β_1_ particles increased to approximately 200–300 nm, but the crystal structure characterization remained unchanged. As shown in the HRTEM image in Figure 5d, the orientation relationship between β′, β_1_, and matrix can also be described as $[1\bar{1}0]\;\beta_{1}//[001]\beta^{\prime }//[0001]\alpha$, $(11\bar{1})\;\beta_{1}//(100)\beta^{\prime }//(11\bar{2}0)\alpha$, and the interplanar spacing distance for β′ and β_1_ phases remains the same as that in the peak-aged samples.

## 4. Discussion

In the present work, the microstructure of the as-cast Mg-7Gd-3Y-2Zn-0.5Zr wt.% (Nd-free) alloy was mainly composed of α-Mg, 14H-LPSO, and 18R-LPSO structures. However, in our previous work, the microstructure of the as-cast Mg-7Gd-3Y-1Nd-2Zn-0.5Zr wt.% (Nd-containing) alloy was composed of α-Mg matrix, (Mg, Zn)_3_RE eutectics, and 14H-LPSO structures [26]. Thus, with the addition of Nd, LPSO structures in the alloy significantly decreased, whereas eutectic (Mg, Zn)_3_RE phases increased, correspondingly. Xingguo Zhang [15] also found that Nd can impede the formation of LPSO phases during solidification. Thus, the formation energy of (Mg, Zn)_3_RE phase may be reduced by the addition of Nd, and the (Mg, Zn)_3_RE phase is inclined to develop Nd-containing alloy during solidification. The formation of (Mg, Zn)_3_RE phase results in the consumption of RE element in the matrix, thus the volume fraction of LPSO phase in a Nd-containing alloy is lower than that in a Nd-free alloy. Aside from the volume fraction, the type of LPSO structure in as-cast alloys is also considerably affected by Nd content. Nd atoms in the alloys prevent the formation of 18R LPSO during solidification. With the addition of Nd, the 18R structure is replaced by 14H LPSO in Nd-containing alloys. Given that the composition of 18R is Mg_10_Y_1_Zn_1_, and Mg_12_Y_1_Zn_1_ is for 14H-LPSO, the RE content in an LPSO structure in a Nd-free alloy is slightly high. Thus, Nd can suppress the formation of (Mg, Zn)_3_RE, but impede the formation of LPSO phases, especially 18R-type during solidification. 

During aging, the significant aging strengthening effect for the Mg–RE–Zn samples can be ascribed to the two types of precipitation phases. These two parallel precipitation sequences consist of the following:

LPSO type: SSSS (h.c.p.) →γ′′→γ′→LPSO (14H + 18R)

Mg-Gd type: SSSS (h.c.p.) →β′′→β′ (b.c.o.)→ β_1_(f.c.c.)→β(f.c.c.)

In this work, these precipitates were found during aging, except for the β′′ and β phases. 

Although the precipitation phases and sequences in Mg-RE-Zn systems have been extensively investigated, some controversies remain with regard to the detailed crystal characterization of these phases, such as the priority of these two types of the precipitation sequence. Jingxu Kent Zheng [27] found that basal γ′′ precipitates serve as the key strengthening phases in an under-aged sample, but β′ formed later during the peak-aged period in Mg–Gd–Ag alloy. Xu [9] reported that Mg–Gd–Y–Zn–Zr alloy aged at both 200 and 225 °C is a supersaturated solid solution (SSSS) (h.c.p.) →β′′→β′. However, stacking faults and 14H LPSO phase formed in the over-aged alloy, which indicates that the precipitation of Mg–Gd type occurred prior to that of the LPSO type, which formed during the over-aged stage. Our previous work on the precipitation behavior of Mg–7Gd–3Y–1Nd–1Zn–0.5Zr [28] reported a similar result: the β′ phase formed in the 18 h aging sample, but single building blocks were first found in the 100 h aging sample. In the present study, the precipitation of the LPSO type was before that of the Mg–Gd type was found in the Nd-free alloy. γ′′ formed in the 18 h aging sample, but the β′ and β_1_ phases precipitated from the matrix until 100 and 200 h, respectively. Thus, for the peak-aged precipitation phases, the priority of these two type sequences was influenced by the Nd element. The nucleation and growth of intermetallic phases was mainly driven by the diffusion control process. Thus, the diffusion behavior of solute atoms can affect the formation of precipitates. Manas Paliwal [29] revealed that the diffusion coefficient of Nd in Mg solid solution is almost three times that of Gd and Y, but lower than that of Al and Zn. In Nd-free alloy, the diffusion rate of Zn atoms is higher than that of Gd and Y. This finding suggests that the LPSO sequence contains Zn precipitated from the matrix. The low solid solubility of Gd and Y in the matrix induced by the formation of LPSO makes Mg–Gd series phases difficult to precipitate until 100 h aging at 240 °C. By contrast, given that the diffusion coefficient of Nd is higher than that of Gd and Y, Mg–Gd series phases form easily in Nd-containing alloys. Thus, the priority of Mg–Gd and LPSO-type sequences changes because of Nd.

Under the peak-age condition, the alloy is mainly strengthened by γ′ and β′/β_1_ precipitates. γ′ is formed on the basal plane, while β′/β_1_ are prismatic precipitates, the habit planes of two type precipitation sequences are perpendicular to each other. During the whole aging process, the β′/β_1_ and γ′/LPSO phases joined together to construct a 3D network. The 3D precipitate network can serve as an obstacle to either *a* or *a+c* dislocation motions, and thus leads to an increase in strength. Thus, to further clarify the strength mechanism of the present alloy, the interactions between these precipitation phases and dislocations that play an important role in the mechanical properties have to be clarified.

**Figure 6:**
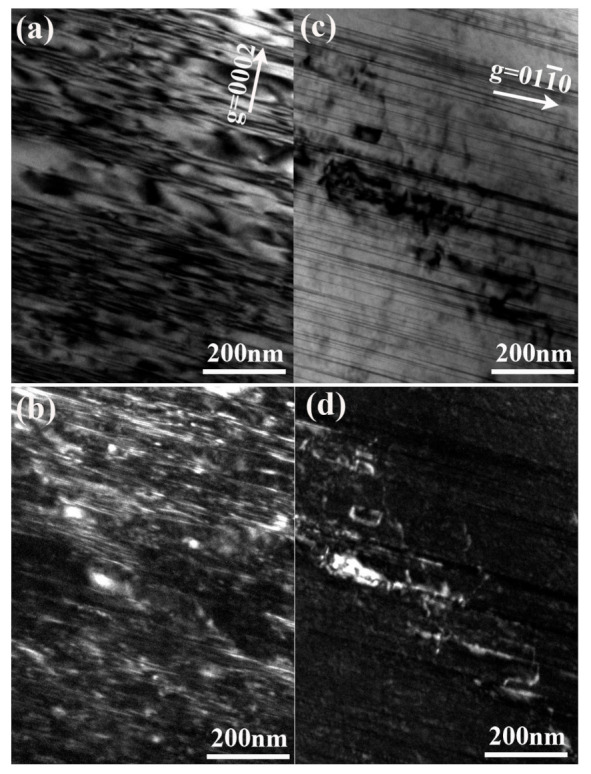
TEM image near the tensile fractured area for 200-h aged alloy: (**a**) bright field(BF) image and (**b**) dark field (DF)image using *g* = 0002; (**c**) BF image and (**d**) DF image using *g* =$\;01\bar{1}0.$

Figure 6 shows the dislocation structures in peak-aged samples. Both bright field and weak-beam dark field images for the same area under different diffraction conditions are presented. In magnesium alloys, it is well known that there are three perfection dislocations, which are *a*, *c*, and *a + c*. On the basis of the *g-b* criterion, the dislocation type in this alloy is judged from its visibility by using *g* = 0002 and *g* = $01\bar{1}0$ in TEM image. Figure 6b,d show the dark-field image of dislocations in as-aged samples near the tensile fracture area. As shown in Figure 6b, many straight *a* dislocations are found on the basal plane, indicating evidence of basal plane slip. When using the *g* = 0002 condition, in which *a* is invisible, some dislocations appear as bright line contrasts, thus indicating that some other type of dislocation exists. Given the complex contrast of high-density dislocations and interfaces between LPSO and the matrix, distinguishing *c* and *c + a* dislocations is difficult. However, such dislocations are regarded as *c + a* dislocations from the deformation mechanism in Mg alloys [30]. Therefore, these dislocations in as-homogenized alloys can be considered *a* dislocations with $\vec{b}=11\bar{2}0\;$ lying on the basal plane. The *a* basal slip was found in both alloys, but *c + a* dislocations are evidence of non-basal slip that developed only in the as-aged alloy. The critical resolved shear stress (CRSS) of non-basal slip systems is much higher than that of basal slip systems at room temperature. However, the activity of *c + a* dislocations after aging indicates that the CRSS of non-basal slip may be decreased by the formation of the LPSO phase. Mitsuhiro Matsuda [30] reported similar findings. Therefore, the LPSO phase formed during aging contributes to the enhancement of the ductility in the present alloy with the increase in the number of the slip system.

## 5. Conclusions

The precipitate phases in an Mg-7Gd-3Y-2Zn-0.5Zr alloy aged isothermally at 240 °C were examined in this work. The aging time to reach peak hardness was about 200 h, and the peak-aged plateau was from 200 h to 500 h. The main conclusions are as follows:

1. The as-cast microstructure of the present alloy was mainly composed of α-Mg, 14H-LPSO, and 18R-LPSO structures. Unlike in our previous Nd-containing alloy, (Mg, RE)_3_Zn eutectic was not found, which indicates that Nd can suppress the formation of (Mg, Zn)_3_RE and impede the formation of LPSO phases, especially 18R-type, during solidification.
2. The two types of precipitation sequence that involve Mg–Gd and LPSO type were found during aging. The LPSO type sequence consists of the precipitation of γ′′, γ′, and 14H-LPSO/18R-LPSO. The Mg–Gd-type precipitation sequence involves the formation of β′(b.c.o.) and β_1_(f.c.c.). The precipitation of the LPSO type occurred prior to that of the Mg–Gd type in the present alloy. The priority precipitation of the Mg–Gd and LPSO type sequences was affected by Nd, which has higher diffusion coefficient than Gd and Y.
3. The coprecipitation of basal γ′ and non-basal β′/β_1_ served as the key strengthening phases at the peak-aged stage. These two types of phases joined to construct a 3D network to strengthen the matrix.
4. The dislocation structure observation suggested that *c*+*a* dislocations developed only in the peak-aged alloy. The activity of *c*+*a* dislocations after aging indicated that the CRSS of non-basal slip may have been decreased by the formation of LPSO phase.

## Data Availability

The data presented in this study are openly available.
